# Hydrogel Encapsulation of *Lactobacillus casei* by Block Charge Modified Pectin and Improved Gastric and Storage Stability

**DOI:** 10.3390/foods10061337

**Published:** 2021-06-10

**Authors:** Qingshen Sun, Louise Wicker

**Affiliations:** 1 Key Laboratory of Microbiology, College of Heilongjiang Province, School of Life Sciences, Heilongjiang University, Harbin 150080, China; sunqingshen@hlju.edu.cn; 2 School of Nutrition and Food Sciences, Louisiana State University Agricultural Center, Baton Rouge, LA 70808, USA; 3 Formerly of Department of Food Science and Technology, University of Georgia, Athens, GA 30605, USA

**Keywords:** charge modified pectin, encapsulation, probiotic, *Lactobacillus*, in vitro release

## Abstract

*Lactobacillus casei (L. casei W8)* was encapsulated in pectin methylesterase (PME) charge modified pectin hydrogels; stability and in vitro release were evaluated under simulated gastrointestinal (GI) conditions. PME, 355 U/mL, de-esterified citrus pectin to 35% from 72% degree of esterification (DE). Pectin ζ-potential decreased to about −37 mV and molecular weight decreased from 177 kDa to 143 kDa during charge modification. More than 99% *L. casei W8* were encapsulated in block charged, low methoxy pectin (35 mLMP) hydrogels by calcium ionotropic gelation. The integrity of the hydrogels was maintained under simulated GI conditions, and no release of *L. casei* W8 was observed. Microbial counts of encapsulated *L. casei* ranged from 6.94 log CFU/g to 10.89 log CFU/g and were 1.23 log CFU/g higher than for unencapsulated *L. casei W8*. The viability of encapsulated *L. casei W8* in wet hydrogels remained the same for 2 weeks, but nearly all flora died after 4 weeks storage at 4 °C. However, freeze dried hydrogels of *L. casei W8* were viable for 42 days at 4 °C and 14 days at room temperature. Charge modified pectin hydrogels are potentially good vehicles for colon-targeted delivery carrier for probiotics and longer stability of *L. casei W8*.

## 1. Introduction

The human gastrointestinal tract is a complex eco-system of microorganisms existing commensally with the human host and influenced by synbiotics, prebiotics, and probiotics [1]. Favorably influencing the gut microbiota is associated with mitigating a myriad of chronic diseases such as obesity, insulin resistance, type 2 diabetes, cancer, and cardiovascular disease, non-alcoholic fatty liver disease, gastrointestinal disorders such as diarrhea, irritable bowel disease, Helicobacter infection [1,2]. In addition to disease treatment, the composition and dynamics of the gut microbiota has the potential to identify risk for disease and the development of personalized nutrition through targeted dietary intervention [3].

For probiotics to exhibit health promoting effects, the viable cells must be at least 7 log CFU after transit through the gastrointestinal tract prior to arrival to the colon [3]. However, probiotics are sensitive to processing and distribution stresses, including heat, light, oxygen exposure, and shear [4,5]. Probiotics are also vulnerable to the harsh conditions in the gastrointestinal tract, such as the extreme peristaltic mechanical action, pH extremes, and bile salts [6].

Of the strategies to protect probiotics during gastrointestinal transit, encapsulation offers significant benefit. Microencapsulated probiotics resist high concentrations of oxygen to ensure high viable count in the production and storage process, resist stomach acid, bile, and digestive enzymes during gastric transit, so that a high number of viable cells potentially colonize the intestinal mucosa. Physical encapsulation techniques include spray drying, freeze drying, spray cooling, complex coacervation, co-extrusion [5]; lipid, protein, and carbohydrate-based entrapment methods include emulsions, solid lipid nanoparticles, nanostructured lipid carriers, liposomes, and layer by layer deposition [4,5].

Hydrogels are an entrapment technique that typically relies on charged carbohydrates and ionotropic gelation for high encapsulation efficiencies [4]. Alginate [7,8] and pectin [9,10] are examples of electrostatic binding and encapsulation via anionically charged carbohydrates. Material for encapsulation also includes blends with other biopolymers, such as protein, including soy protein isolate-high methoxyl pectin microcapsules [11], and whey [12,13]. Excellent reviews on encapsulation of probiotics include a summary of critical issues and impact of the large size of microbial cells as a limiting factor in the choice of encapsulation methods [14] and describes stability of pectin hydrogels alone or including chitosan, rice bran, or whey [15].

In addition to ionotropic gelling properties, pectin is a versatile plant polysaccharide with diverse structure and multiple health benefits [16,17,18]. Pectic fractions also show prebiotic activity and stimulates in situ growth of probiotics [19]. Low methoxyl pectin (LMP) readily reacts with cations, such as calcium, and is typically used in colon-targeted drug delivery system [20]. Encapsulated addenda include probiotics, antioxidants, enzymes, or other bioactives [21,22].

The total charge, as well as the distribution of carboxylic acid groups affects pectin gelling properties and block-wise rather than random de-esterification of pectin results in stronger gelling properties [23,24,25,26,27,28]. Pectin methylesterase isozymes influence the extent and pattern of de-esterification [24,25,26,29] and calcium pectate gel strength increases non-linearly with de-esterification [28]. Block de-esterified high methoxy pectin was a more effective encapsulant of indomethacin than randomly de-esterified pectin and under simulated gastric conditions in vitro, encapsulated indomethacin with block de-esterified pectin showed <1% release [30].

In this study, a pectin methylesterase extracted from citrus pulp was used to charge modify pectin in a block-wise, sequential distribution of carboxylic acid groups to low methoxyl pectin. The charge modified pectin was used to encapsulate *L. casei* W8. The in vitro tests were performed to verify the possibility of using pectin hydrogels as a carrier for colon-targeted probiotic delivery system. The objectives of this study were to evaluate block charge modified citrus pectin for encapsulation efficiency of probiotic bacteria, to evaluate the probiotic stability under simulated gastrointestinal conditions, and to evaluate the probiotic stability during storage.

## 2. Materials and Methods

### 2.1. PME Activity and Pectin Modification

PME was extracted from Valencia citrus pulp (donated by Citrus World, Lake Wales, FL, USA) by homogenization in 0.1 M sodium chloride and 0.25 M Tris-HCl, pH 8.0 at 4 °C, as described earlier [31]. Following ammonium sulfate precipitation and dialysis against 0.05 M sodium phosphate buffer, pH 7.0, the dialyzate was used as PME extract.

PME activity was measured using a pH stat titrator (Brinkmann, Westbury, NY, USA) as described previously [31]. An aliquot of 1% high methoxyl pectin, (GENU pectin type B rapid set-Z from citrus peel, DE 72%, Batch NO: GR41649, CP Kelco, Copenhagen, Denmark) was prepared in deionized water with 0.1 M sodium chloride and hydrated overnight, 4 °C. After adjusting the temperature of the pectin dispersion to 30 °C, an aliquot of 25 μL PME was added and the amount of standardized sodium hydroxide needed to maintain the pH at 7.5 was recorded during the time of the assay. The PME activity of 355 U/mL was expressed as µequivalents of ester hydrolyzed per minute at 30 °C and pH 7.5.

Pectin was charge modified to a target degree of esterification of 35% (35 mLMP) as described previously [32]. An aliquot of 80 g HMP was dispersed in 4 L 0.1 M NaCl to make 2% pectin, and hydrated overnight at 4 °C. After pectin equilibration to 30 °C, pectin charge modification was initiated by addition of known units of PME activity, to achieve the desired de-esterification to 35% DE in 20 min, pH 7.5. Immediately at the end of the 20 min, the pH was adjusted to pH 5.0 to stop PME activity; the mixture was poured into boiling ethanol and agitated vigorously. The mixture was boiled for 10 min to inactivate PME and cooled to room temperature. The mixture was filtered using Mira-cloth (EMD Millipore Corporation, Billerica, MA, USA) and the pellet was washed once with acetone, before drying under the hood on a glass plate. The control samples were treated with the same procedure except that buffer was added in lieu of PME.

### 2.2. Characterization of 35 mLMP

Particle size and zeta potential determination. Particle size and zeta potential of control and PME treated pectin was measured, using a Particle Size Analyzer (90 Plus, Brookhaven Inst., Holtsville, NY, USA) with a 50 mW diode laser and a BI-9000AT correlator [33]. Pectin at 5 mg/mL was dissolved in the 0.01 M phosphate sodium buffer at room temperature and hydrated overnight at 4 °C; dispersions were filtered through 5 µm Millex^®^-SV filters (low protein binding Durapore^®^ membrane, Lot: R4CA22792, Merck Millipore Ltd., Darmstadt, Germany), and diluted to 1 mg/mL using the 0.01 M sodium phosphate, pH 7.4. All experiments were carried out at 25 °C with the laser beam at 659 nm and refractive index of 1.330. The particle size was recorded for zeta potential and particle size were determined in triplicate.

For gel permeability chromatography analysis, 3 mg/mL pectin samples were prepared 10 mM sodium phosphate and 100 mM sodium nitrate, pH 7, and filtered through 13 mm, 25 µm PES filter (Whatman, Maidstone, UK) before injection. Dispersions were analyzed using the HPSEC–multi-angle light scattering system composed of an Infinity 1260 isocratic pump with a degasser (Agilent Technologies, Santa Clara, CA, USA), a SpectraSYSTEM AS1000 auto sampler (Thermo Fisher Scientific Inc., Waltham, MA, USA), a PL-Aquagel-OH mix column (300 × 7.5 mm) with PL-Aquagel-OH guard column (50 × 7.5 mm) (Agilent Technologies, Santa Clara, CA, USA), a Dawn Heleos-II multi-angle light scattering (MALS) detector (Wyatt Technology Corporation, Santa Barbara, CA, USA), and an Optilab T-rEX differential refractive index (RI) detector (Wyatt Technology Corporation, Santa Barbara, CA, USA). The flow rate was set at 0.5 mL/min, with 10 mM sodium phosphate, and 100 mM sodium nitrate, pH 7 as eluent buffer [34]; analysis was conducted with ASTRA software (Version 6.1, Wyatt, Santa Barbara, CA, USA).

### 2.3. Encapsulation of L. casei W8

The strain of lactic acid bacteria, *L. casei W8*, was donated by Chr. Hanson HØrsholm, Denmark and stored at −80 °C. Buffered peptone water (VWR Catalog Number: 89405-868, Atlanta, GA, USA) was prepared and autoclaved at 121 °C for 20 min. The *L. casei W8* granulated powder was dissolved in buffered peptone water, at a ratio of 1:2 (*w*/*v*). The dispersions of *L. casei W8* and 2% pectin were mixed in equal ratio (*w*/*w*). For control, the same amount of peptone water was added to 2% pectin dispersion in lieu of added *L. casei W8*.

**Figure 1:**
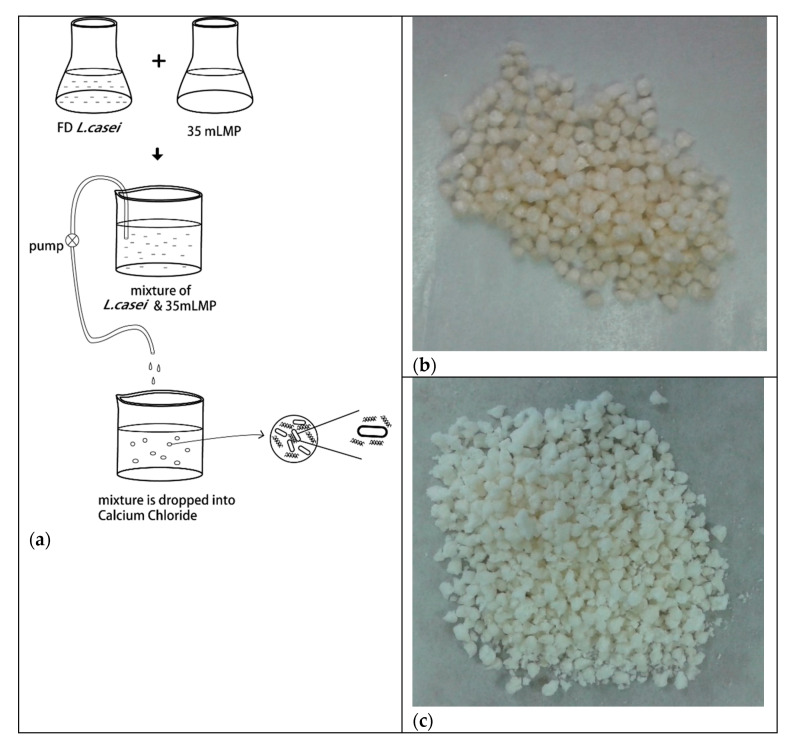
(**a**) Process diagram to prepare encapsulated *Lactobacillus casei* in pectin hydrogels. (**b**) Image of freeze-dried beads, mLMP beads without cryoprotectant; (**c**) mLMP beads with protectant.

The pectin hydrogels were prepared by ionotropic gelation [30]. In brief, 2% pectin (*w*/*v*) with *L. casei W8* was extruded into 300 mM calcium chloride while stirring, gently stirred for 20 min, and equilibrated without stirring for 4 h at room temperature. The hydrogels were filtered through Mira-cloth and divided into two parts to be stored at 4 °C or freeze-dried. The wet beads were stored in a beaker and covered with parafilm prior to storage at 4 °C. See Figure 1 for a schematic drawing of the encapsulation process.

Hydrogels were disintegrated with calcium chelators to estimate encapsulation efficiency. The hydrogels were dissolved in an autoclaved solution of 50 mM EDTA and 50 mM ammonium oxalate. Upon disintegration, the mixture was centrifuged at 3928× *g* for 15 min at 4 °C and the pellets were collected; pellets were re-suspended in 0.9% saline and centrifuged again to remove residual chelator and diluted for plate counting.

MRS agar was prepared according to manufacturer’s guide and autoclaved at 121 °C. After cooling to 50 °C, the media was poured into 10 cm plates and cooled. The collected flora was serially diluted and spread onto the plates. After anaerobic culture at 37 °C for 40–44 h, the *L. casei W8* were counted and calculated as log CFU/mL (for supernatants) or log CFU/g (for hydrogels). For enumeration of the *L. casei W8i* in supernatants, the supernatant was diluted directly and spread on pates.

The encapsulation efficiency (EE) was calculated based on plate counting results:

EE = (A − B)/A

where A and B represent the total number of added *L. casei W8* and measured cells in the supernatants, respectively.

### 2.4. Stability and Release of Probiotic In Vitro

The cryoprotectant was composed of 10.43 g skim milk, 8.91 g glucose, and 0.23 g MnSO_4_ in 100 mL sterilized water, which was mixed and boiled at 100 °C for 10 min, then cooled to 4 °C. The hydrogels were mixed with cryoprotectant at 1:1 (*w*/*v*) [35]. The mixture was placed at −20 °C for 30 min and freeze dried for 40–44 h. One part of the freeze-dried cells was stored in a desiccator at room temperature and another part was stored at 4 °C in refrigerator. To determine the survival rate after freeze-drying, disintegration of pectin hydrogels was performed as described above. The number of the viable flora was quantified by plate counting.

Simulated gastric fluid (SGF) was composed of 0.03 M NaCl solution containing 1.6 g pepsin in 500 mL deionized water; the pH was adjusted to 2.0 with 10 N HCl. Simulated bile fluid (SBF) was prepared by adding 1 g porcine bile extract into 100 mL 0.2 M phosphate, pH 7.4. Simulated intestinal fluid (SIF) was prepared by adding 2 g pancreatin into 400 mL 0.2 M phosphate (pH 7.4) as described earlier [36].

In vitro release tests were performed by placing the free *L. casei W8* and hydrogels in SGF for 2 h, SBF 20 min, or SIF 3 h, or sequential SGF-SBF-SIF at the same time periods [36]. After each process, the hydrogels were collected and disintegrated using 50 mM ammonium oxalate and 50 mM [36] (EDTA-OA), as described earlier. The collected flora was diluted and enumerated on MRS plates.

The stability of *FD L. casei* was estimated after storage of *L. casei*, *L. casei* with skim milk, *L. casei* hydrogels, and *L. casei* hydrogels with skim milk at 4 °C for 0, 7, 14, and 28 d, followed by enumeration by plate counting as described above. Moreover, the freeze-dried hydrogels were stored at 4 °C or room temperature and tested at 7, 14, 28, and 42 d in triplicate.

### 2.5. Statistical Analysis

PME charge modified pectin was prepared twice and three independent batches of *FD L. casei W8* loaded pectin hydrogels were prepared. The results reported were the mean values obtained from these three batches. All data was analyzed for significant differences (*p* = 0.05), using Origin 8.0 software (Origin Lab Corporation, Northampton, MS, USA). The results were expressed as mean ± standard deviation.

## 3. Results

### 3.1. Charge Modification and Characterization of Pectin

In this study, charge modified pectin was prepared by block-wise, enzymatic de-esterification, characterized and evaluated for encapsulation efficiency and stability of the probiotic culture; the physico-chemical characterization of the starting citrus pectin, with a 72% degree of esterification and the two preparations of PME modified pectin used for encapsulation and stability studies are reported in Table 1. The initial ζ-potential was −27 ± 0.1 to −31 ± 1.0 mV and decreased to −36 ± 0.8 to −37 ± 1.5 mV after charge modification. The removal of the methyl ester increased the anionic nature of pectin as evidenced by the more negative zeta potential of the dispersions. The ζ-potential of 35 mLMP is consistent with the ζ-potential values of block de-esterified pectin [28,32].

Particle size of 72 HMP control ranged from 577 ± 11 to 614 ± 14 nm and decreased negligibly to 546 ± 11 to 585 ± 21 nm for 35 mLMP. In HMP control, a molecular weight of 171 to 177 kDa and polydispersity value above 1.8 were observed. In 35 mLMP, molecular weight decreased to about 142 kDa polydispersity value of 1.7. The decrease in molecular weight is likely attributed to β-elimination due to extended times at alkaline pH values [37]. Localized hydrolysis and saponification were especially problematic resulting from poor mixing in pectin dispersions at higher concentrations. The high polydispersity values were reflective of the heterogeneity of pectin, with molecular weight values that range from 10 to over 500 kDa [17]. Molecular weight did not change during charge modification [32], which may be explained by the smaller change in target DE value, from 73% to 63% or 61%. Plant PMEs, such as citrus PME, created blocks of charge that facilitate the ion-crosslinking of 35 mLMP with calcium chloride [33].

### 3.2. Encapsulation and Simulated Gastrointestinal Release

Chelators were used to disrupt the encapsulated *L. casei* beads for enumeration and the viability of the encapsulated microbes was determined by plate counts after EDTA and EDTA plus ammonium oxalate treatment. No significant difference was observed between EDTA and EDTA plus ammonium oxalate (OA) treatment with recovery counts between 2.9 × 10^11^ and 4.1 × 10^11^ CFU/g, respectively (data not shown). However, EDTA plus OA treatment expedited the disintegration of the beads. Therefore, the EDTA-OA solution was used in all subsequent disintegration tests.

Encapsulation efficiency was high with a 4 to 6 log CFU decrease of the *L. casei W8* in supernatants from hydrogels prepared with 35 mLMP, indicating that encapsulation efficiency was higher than 99%. The *L. casei W8* survival number after encapsulation with 35 mLMP hydrogels was 11.06 ± 1.34 log CFU/g (on dry *L. casei W8* capsules basis). The control, unmodified pectin, 72 HMP, did not form hydrogels with calcium chloride, due to insufficient charge on pectin, and was not evaluated further.

The stability and in vitro release profile in simulated gastrointestinal fluids is summarized in Table 2. The stability of *L. casei* was not affected (*p* > 0.05) by the encapsulation process, nor by freeze-drying with skim milk cryoprotectant. A 3-log CFU/g beads decrease was observed in freeze-dried beads with no cryoprotectant. The unencapsulated, *L. casei W8* was vulnerable to SGF and SBF, and counts decreased (*p* < 0.05) from 11.24 ± 0.81 to 4.23 ± 0.25 and 0.89 ±0.44 log CFU/g beads, respectively. In SIF, microbial numbers remained at 11.65 ± 0.22 log CFU/g beads, indicating that *L. casei W8* was stable under small intestinal or colonic conditions only if the higher survival rate can be obtained without destruction by gastric acid or bile salt. After sequential SGF-SBF-SIF treatment, log CFU/g beads of 1.23 ± 0.32 was measured, showing sensitivity to SGF and SBF, primarily. To further test the potential colonic viability of *L. casei W8,* the unencapsulated and encapsulated hydrogels, which had been treated sequentially with SGF-SBF-SIF, were incubated for 15 h at 37 °C anaerobically. The encapsulated *L. casei W8* showed 5 log CFU/mL higher counts compared to those without hydrogel encapsulation under colonic conditions, suggesting potentially complete release of the encapsulated *L. casei W8* in the colon.

### 3.3. Stability Studies

After the survival rate of wet hydrogels under simulated gastrointestinal conditions was determined, it was important to know the survival rate of *L. casei W8* when stored at 4 °C as wet hydrogels, with or without cryoprotectants. Maintaining high counts during storage and prior to use ensured a viable dose of probiotic to the gastrointestinal system. The stability of wet hydrogels during storage for 28 d at 4 °C is depicted in Table 3. All initial counts were above 11.80 log CFU/g. At day 7, *L. casei* and *L*. *casei* with 35 mLMP had the lowest counts at 2.32 and 4.32 log CFU/g, respectively. Skim milk without or with 35 mLMP resulted in 10.87 and 11.94 log CFU/g, respectively. By day 14, there were no viable counts in the control *L. casei* and greatly reduced counts in all but *L. casei* with the combined cryoprotectants of skim milk and 35 mLMP. By day 28, only 35 mLMP hydrogels loaded with *L. casei W8* mixed with skim milk cryoprotectant had viable counts (10.39 ± 0.06 log CFU). For wet hydrogels stored at 4 °C for 28 days without freeze drying, no growth was found except *L. casei W8* encapsulated hydrogels with cryoprotectant, indicating that high humidity and oxygen damages the unprotected *L. casei W8* (Table 3). Clearly, for sustained stability of *L. casei*, encapsulation with 35 mLMP and inclusion of skim milk was beneficial.

When *L. casei W8* loaded hydrogels were freeze-dried and stored at 4 °C, stability was higher than when stored as wet hydrogels (Table 4). At 42 days, all treatments had viable, albeit reduced viable flora when stored at 4 °C. At 42 days, 35 mLMP hydrogels loaded with *L. casei W8* mixed with cryoprotectant, had 12.98 ± 0.010 at day 0 and 10.48 ± 0.017 CFU/ g beads at day 42 (*p* < 0.05). Viable flora was less when hydrogels were stored at room temperature (Table 4). Notably, *L. casei W8* loaded hydrogels with 35 mLMP, at room temperature, had highest counts at 28 days compared to other cryoprotectants, but after 7 days, counts were below the desired threshold.

## 4. Discussion

The PME charge modified pectin, 35 mLMP, with low DE and negative zeta potential, encapsulated, and protected *L. casei* W8. Unlike commercially prepared low methoxyl pectin, with random de-esterification, citrus PME m35LMP resulted in block-wise de-esterification of pectin, which when chelated with calcium, results in stronger, more rigid gel formation [23,25]. Pectin hydrogel strength of pectins with DE values between 36% and 40% ranged between 748 to 584 Pa [28]. Furthermore, the pattern of de-esterification of PME isozymes influences gel strength [33] and block-wise de-esterified pectin with more rigid gels, had greater encapsulation and slower release than randomly de-esterified pectin [30]. The dimensions of probiotic bacteria and pectin are measured in µm and nm, respectively [38,39]. The formation of the pectin hydrogel network around the large probiotic bacteria likely involves direct adsorption of some pectin onto the probiotic surface, the formation, and extension of intermolecular calcium pectate strands as well as formation of a three-dimensional network structure of calcium pectate between multiple pectin chains (Figure 1). The strong, rigid gel structure immobilizes the probiotic and creates a barrier resistant to diffusion of small molecules, such as gastric and intestinal fluids.

Light, oxygen, and water all contribute to the instability of the encapsulated *L. casei* W8. The charge modified pectin, 35 mLMP, had more than 99% encapsulation efficiency for *L. casei W8*. The viability of *L. casei* W8 as wet hydrogels was maintained for 2 weeks, with 2 log CFU decrease at the second week. For freeze-dried hydrogels, 4 °C storage was better than storage at room temperature. The stability of probiotics under simulated gastric or intestinal conditions indicated that the freeze-dried or wet beads had less stability under gastric conditions but nearly 90% retention was measured with encapsulated L. casei under sequential simulation of gastric, bile, and intestinal conditions. Storage and simulated gastrointestinal stability after encapsulation of lactic acid bacteria in other polymers or copolymers, such as alginate was highly effective with viability above 8 CFU/g in a mixed probiotic culture [40] While L. casei ATCC 393 cells encapsulated in soy protein isolate-alginate hydrogels had similar stability under storage or simulated gastrointestinal conditions compared to non-encapsulated cells [41] soy protein isolate/sugar beet pectin hydrogels of *L. paracasei* LS14 showed improved storage and simulated gastrointestinal stability [42] Encapsulated L. plantarum cells in pectin–starch hydrogels were more stable than free cells during storage and simulated gastrointestinal conditions with nearly 7 CFU/g retention [43] Higher stability in pectin–chitosan hydrogels of L. casei were attributed to the basic chitosan layer over pectin, preventing acid infiltration during gastric simulation [44] In the present study, the hydrogel encapsulated *L. casei W8* showed good colonic-targeted release potential using only charge modified pectin as the encapsulating agent. The enhanced effectiveness of charge modified pectin is likely due to the formation of numerous, strong junction zones, that immobilize the lactic acid bacteria in are multiple layers of a pectin gel network and prevent diffusion of simulated intestinal fluids. In a companion study, *L. casei W8*, encapsulated by charge modified pectin was evaluated for physiological responses of a high fat diet in a rat feeding trial; *L. casei* W8 changed the gut microbiota [45]. The free *L. casei W8* improved gut barrier properties and reduced systemic and localized inflammation in high fat fed rats; encapsulated *L. casei W8* improved glucose tolerance [45], providing evidence for the beneficial physiological effect in a high fat diet of free and encapsulated *L. casei W8.*

## 5. Conclusions

Commercially available, high methoxyl pectin can be readily charge-modified to increase total charge and increase contiguous blocks of charge. The charge modified pectin shows high encapsulation efficiency and encapsulated probiotics show higher, longer-term stability under gastrointestinal simulations, especially when freeze-dried with cryoprotectant. The high encapsulation efficiency and stability using charge modified pectin hydrogels are sufficient to ensure colonic delivery of a sufficient number of viable cells. Health outcomes associated with probiotic bacteria are likely enhanced by the higher number of viable cells.

## Figures and Tables

**Table 1:** Zeta potential, particle size, molecular weight, and polydispersity of pectin samples.

	Sample	ζ-Potential (mV)	Particle Size (nm)	Molecular Weight (kDa)	Polydispersity (Mw/Mn)
Trial 1 ^1^	35 mLMP ^2^	−37 ± 1.5	585 ± 21	143 ± 2.0	1.7 ± 3.0
	72 HMP control ^3^	−27 ± 0.1	614 ± 14	177 ± 2.0	1.8 ± 2.9
Trial 2	35 mLMP ^2^	−36 ± 0.8	546 ± 11	141 ± 2.2	1.7 ± 3.3
	72 HMP control ^3^	−31 ± 1.0	577 ± 11	171 ± 2.1	2.0 ± 3.3

^1^ Trial 1 and Trial 2 refer to duplicate charge modified pectin preparations used to have sufficient material to conduct analysis; ^2^ 35 mLMP was pectin treated with 25 µL pectin methylesterase, 355 Units/mL; ^3^ 72 HMP control was pectin treated exactly as treatment 35 mLMP pectin except 25 µL buffer was added in place of pectin methylesterase.

**Table 2:** In vitro simulated gastrointestinal tract test of Lactobacillus stability (log CFU/g bead).

ORIGINAL	SGF 2 H	SBF 20 MIN	SIF 3 H	SGF-SBF-SIF
*Lactobacillus casei* W8 CONTROL				
11.24 A ± 0.81	4.23 ^A^ ± 0.25	0.89 ^A^ ± 0.44	11.65 ^A^ ± 0.22	1.23 ^A^ ± 0.32
WET *Lactobacillus casei* W8 BEADS				
12.94 A ± 0.12	10.86 ^B^ ± 0.10	9.90 ^B^ ± 0.09	12.47 ^A^ ± 0.08	10.89 ^B^ ± 0.32
Freeze-Dried *Lactobacillus casei* W8 Beads—No Skim Milk				
7.81 B ± 0.24	7.30 ^C^ ± 0.01	7.12 ^C^ ± 0.89	7.37 ^B^ ± 0.69	6.94 ^C^ ± 0.96
Freeze-Dried *Lactobacillus casei* W8 Beads—With Skim Milk				
10.16 A ± 0.54	9.26 ^D^ ± 0.07 ^D^	9.78 ^B^ ± 0.32	12.00 ^A^ ± 0.24	9.03 ^D^ ± 0.82

Acronyms are denoted by: SGF, simulated gastric fluid; SBF, simulated bile fluid; SIF, simulated intestine fluid; SGF-SBF-SIF refers to sequential SGF treatment for 2 h, SBF for 20 min and SIF for 2 h 40 min. Different uppercase letters in the same column represent a significant difference, *p* < 0.05. All flora were counted on dry lactobacillus basis.

**Table 3:** Stability studies on the wet *Lactobacillus casei* W8 beads stored at 4 °C.

SAMPLE	0 DAY	7 DAYS	14 DAYS	28 DAYS
*L. casei* W8	12.91 ^B^ ± 0.009	2.32 ^D^ ± 0.025	ND	ND
Skim Milk, *L. casei* W8	11.95 ^C^ ± 0.008	10.87 ^B^ ± 0.078	6.64 ^B^ ± 0.031	ND
mLMP, *L. casei* W8	11.80 ^D^ ± 0018	4.32 ^C^ ± 0.006	3.11 ^C^ ± 0.026	ND
Skim Milk, mLMP, *L. casei* W8	12.99 ^A^ ± 0.008	11.94 ^A^ ± 0.008	10.45 ^A^ ± 0.096	10.39 ± 0.057

Note: all viable flora were calculated based on the number of Lactobacillus on dry weight base (CFU/g) for comparison as the initial viable Lactobacillus was calculated around 10^12^ CFU/g. “ND” represents no viable flora were found. Different uppercases represent the significant difference at *p* < 0.05 level in the same column. “ND” indicates no viable flora were found.

**Table 4:** Stability studies on the freeze-dried beads of *Lactobacillus casei* W8 stored at 4 °C and room temperature.

	**4 °C**			
**Day**	***L. casei* W8**	**Skim milk, *L. casei* W8**	**mLMP, *L. casei* W8**	**Skim milk, mLMP,** ***L. casei* W8**
0	11.92 ^A^ ± 0.002	11.94 ^A^ ± 0.008	7.82 ^A^ ± 0.005	12.98 ^A^ ± 0.010
7	11.81 ^A^ ± 0.004	11.92 ^A^ ± 0.003	7.48 ^C^ ± 0.032	12.89 ^A^ ± 0.011
14	11.50 ^B^ ± 0.015	10.68 ^B^ ± 0.010	7.33 ^C^ ± 0.028	11.82 ^B^ ± 0.704
28	11.48 ^B^ ± 0.013	9.18 ^C^ ± 0.722	7.63 ^B^ ± 0.020	11.52 ^B^ ± 0.028
42	10.98 ^C^ ± 0.111	8.21 ^D^ ± 0.058	7.61 ^B^ ± 0.008	10.48 ^C^ ± 0.017
	**Room Temperature**			
**Day**	***L. casei* W8**	**Skim milk, *L. casei* W8**	**mLMP, *L. casei* W8**	**Skim milk, mLMP,** ***L. casei* W8**
0	11.92 ^A^ ± 0.002	11.94 ^A^ ± 0.008	7.82 ^A^ ± 0.005	12.98 ^A^ ± 0.010
7	2.91 ^B^ ± 0.012	11.94 ^A^ ± 0.000	7.83 ^A^ ± 0.020	12.99 ^A^ ± 0.006
14	0.89 ^C^ ± 0.269	1.10 ^B^ ± 0.213	6.51 ^B^ ± 0.019	6.03 ^B^ ± 0.029
28	ND	ND	6.38 ^B^ ± 0.085	2.59 ^C^ ± 0.042
42	ND	ND	ND	ND

Note: viable flora number was calculated as the number of *Lactobacillus casei* W8 on dry basis (CFU/g) for comparison as the initial viable Lactobacillus was calculated around 10^12^ cfu/g. “ND” indicates no viable flora were found. Different uppercases represent the significant difference at *p* < 0.05 level in the same column.
